# Determination of a distinguished interferon gamma epitope recognized by monoclonal antibody relating to autoantibody associated immunodeficiency

**DOI:** 10.1038/s41598-022-11774-9

**Published:** 2022-05-09

**Authors:** Umpa Yasamut, Tanchanok Wisitponchai, Vannajan Sanghiran Lee, Montarop Yamabhai, Kuntalee Rangnoi, Weeraya Thongkum, Koollawat Chupradit, Chatchai Tayapiwatana

**Affiliations:** 1 Division of Clinical Immunology, Department of Medical Technology, Faculty of Associated Medical Sciences, Chiang Mai University, Chiang Mai, Thailand; 2 Center of Biomolecular Therapy and Diagnostic, Faculty of Associated Medical Sciences, Chiang Mai University, Chiang Mai, Thailand; 3 Center of Innovative Immunodiagnostic Development, Faculty of Associated Medical Sciences, Chiang Mai University, Chiang Mai, Thailand; 4 Department of Chemistry, Faculty of Science, University of Malaya, Kuala Lumpur, Malaysia; 5 Molecular Biotechnology Laboratory, School of Biotechnology, Institute of Agricultural Technology, Suranaree University of Technology, Nakhon Ratchasima, Thailand

**Keywords:** Computational biology and bioinformatics, Immunology

## Abstract

Anti-interferon gamma autoantibodies (anti-IFN-γ autoAbs) neutralize the IFN-γ-mediated functions, contributing to immunodeficiency. A particular autoAb in patient serum had been previously demonstrated to recognize the same determinant on IFN-γ as the neutralizing anti-IFN-γ monoclonal antibody clone B27 (B27 mAb). This study explored the epitope recognized by B27 mAb. The specific peptide sequence recognized by B27 mAb, TDFLRMMLQEER, was retrieved from a phage display random peptide library. Sequence alignment and homology modeling demonstrated that the queried phage peptide sequence and structure were similar to amino acids at position 27–40 (TLFLGILKNWKEES) of the human IFN-γ. This determinant resides in the contact surface of IFN-γ and interferon gamma receptor 1. To elucidate the crucial amino acids, mutations were introduced by substituting T27 and T27F29L30 with alanine or deleting the amino acid residues T27–L33. The binding of B27 mAb to IFN-γ T27A using western blotting was lesser than that to wild-type. The interaction with triple mutant and T27–L33 deletion mutant using western blotting and sandwich ELISA was abolished. The finding demonstrated that T27, F29, and L30 are critical residues in the B27 antigenic determinant. Identification of the functional domain of IFN-γ decrypted the relevance of neutralizing autoAb in adult-onset immunodeficiency.

## Introduction

Interferon gamma (IFN-γ) is a type II interferon that plays pleiotropic roles in the innate and adaptive immune system^1^. It demonstrates anti-viral and anti-mycobacterial activity, antigen presentation by upregulation of major histocompatibility complex (MHC) molecules, anti-proliferative effects, and immunosuppression^2^. Structurally, IFN-γ is a homodimer, consisting of a non-covalent self-assembly in a head-to-tail orientation. The helical regions A and B with their connecting loop, a histidine residue at position 111 (H_111_) in the F helix, and the flexible C terminus are important regions for receptor binding^3^. Ligand binding results in receptor oligomerization, with two α-receptor chains, IFN-γR1, bound to one IFN-γ homodimer, followed by recruitment of two β-receptor chains, IFN-γR2, to the complex inducing the expression of IFN-γ-stimulated genes^4,5^.

The presence of neutralizing anti-IFN-γ autoAbs is associated with adult-onset immunodeficiency (AOID)^6–10^. Patients lacking IFN-γ-mediated functions are susceptible to opportunistic infections, especially nontuberculous mycobacterial (NTM) infections. In 2016, Lin et al. identified an epitope recognized by anti-IFN-γ autoAbs using 30-mer non-overlapping synthetic peptides. The data illustrated the C-terminal region of IFN-γ (amino acid 121–131, SPAAKTGKRKR) as a sequential epitope recognized by the patient’s autoAbs^11^. Recently, the neutralizing autoAb recognizing discontinuous epitope in patients with mycobacterial infection was identified (Patent No. WO 2018/202200 A1). However, the epitope recognized by the pathogenic autoAbs has not yet been thoroughly investigated. In a previous study, we had reported that autoAbs in patients with AOID competed with neutralizing mouse anti-IFN-γ monoclonal antibody (mAb) (clone B27)^12^. Epitope mapping using 20-mer synthetic peptides revealed that B27 mAb does not bind to the C-terminal epitope.

Recently, the conformational epitopes recognized by other neutralizing anti-IFN-γ mAbs have been identified using human-bovine chimeric proteins. Accordingly, two major epitopes, including regions A and E, were discovered^13^. Moreover, regions A and E-recognizing mAbs displayed various degrees of neutralizing activity. This finding confirmed that IFN-γ is composed of various conformational epitopes. Among the epitope mapping tools, phage display random peptide library is a powerful technique for epitope determination. Previously, this technique has been used to identify the epitopes of anti-TNF-α autoAbs in patients with rheumatoid arthritis (RA)^14^. Results had shown that the identified peptides inhibited the binding of autoAb to TNF-α. In our previous effort to identify the epitope recognized by B27 mAb, it failed to interact with overlapped synthetic peptides across the IFN-γ sequence. Accordingly, we applied a phage display random peptide library to further discover this epitope. In addition, structural analysis and homology modeling were coordinated to elucidate the key amino acid residues participating in this epitope. The candidate residues from ^27^TLFLGILKNWKEES^40^ were proposed for further mutations. Disclosure of the novel epitope relating to anti-IFN-γ autoAbs in patients will provoke the study of the molecular pathology of AOID.

## Results

### Epitope mapping by phage display random peptide library

From eight randomly picked phage clones, six showed positive binding in phage ELISA (data not shown). DNA sequence analysis indicated them to be the same phage clones, displaying the peptide sequence “TDFLRMMLQEER”. Since only one amino acid sequence was obtained, eight more phage clones were randomly picked. Two additional phage clones that showed positive binding in phage ELISA were sent for DNA sequence analysis and the same amino acid sequence was identified once again. The data suggested that B27 mAb is specific in recognizing this peptide sequence.

### Sequence alignment

Sequence alignment demonstrated that the queried phage peptide sequence coincides with a portion located at ^27^TLFLGILKNWKEES^40^ of the human IFN-γ with 35.71% identity and 57.14% similarity. Non-homology gaps were at positions 34 and 35.

### Location of the peptide on human IFN-γ structure

**Figure 1:**
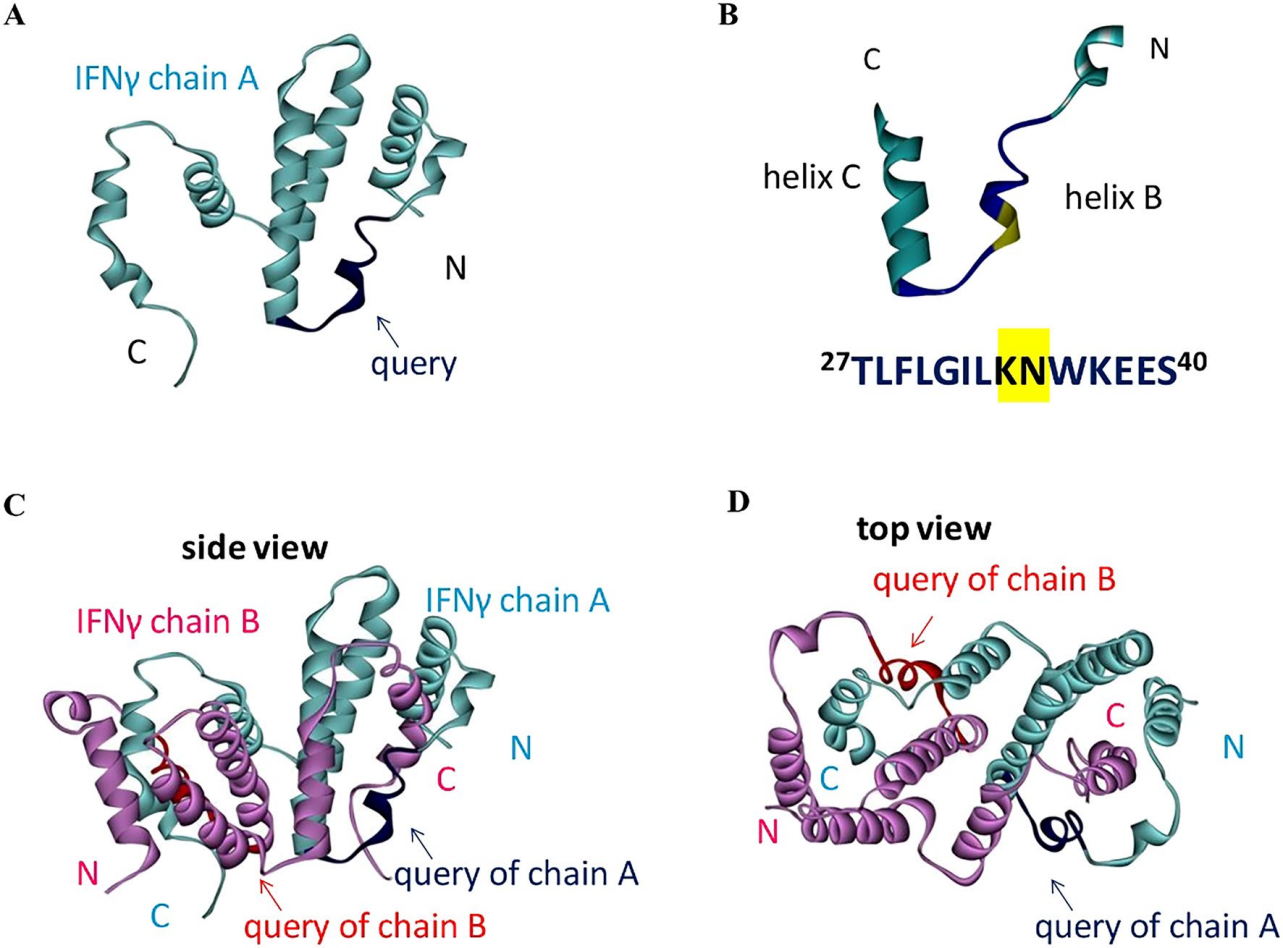
The query portion on human IFN-γ structure, PDB ID: 1FG9. (**A**) Single IFN-γ molecule, (**B**) the gap position on IFN-γ (yellow-highlight), (**C**) side view of homo-dimer IFN-γ, and (**D**) top view of homo-dimer IFN-γ. Blue and red colors represent the query aligned on IFN-γ chain A and chain B, respectively.

**Figure 2:**
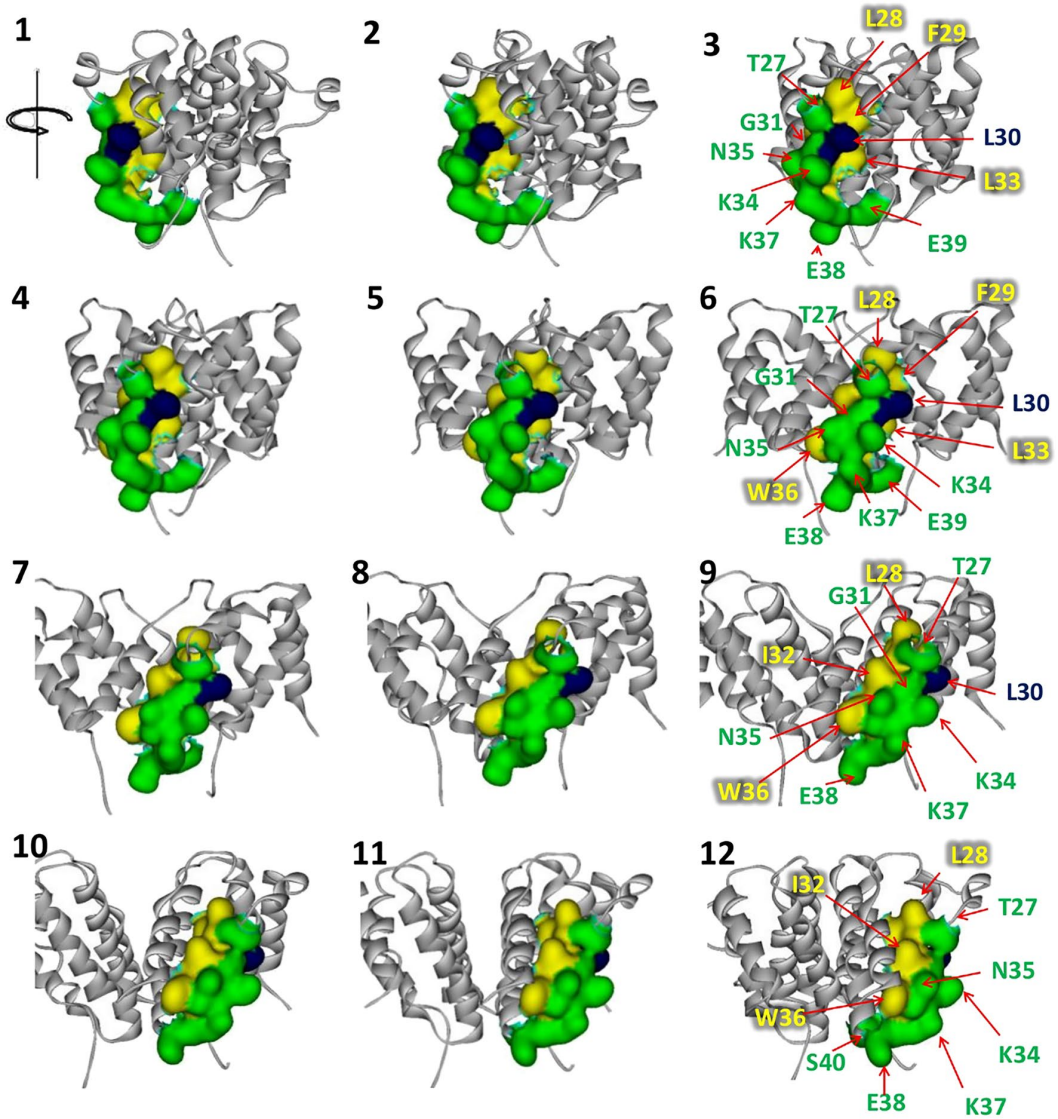
Rotation view of solvent accessible residues of ^27^TLFLGILKNWKEES^40^ in human IFN-γ. The buried (yellow), exposed (green), and intermediate (blue) residues were classified with respect to % SASA, < 10%, > 25%, and 10%–25%, respectively.

**Figure 3:**
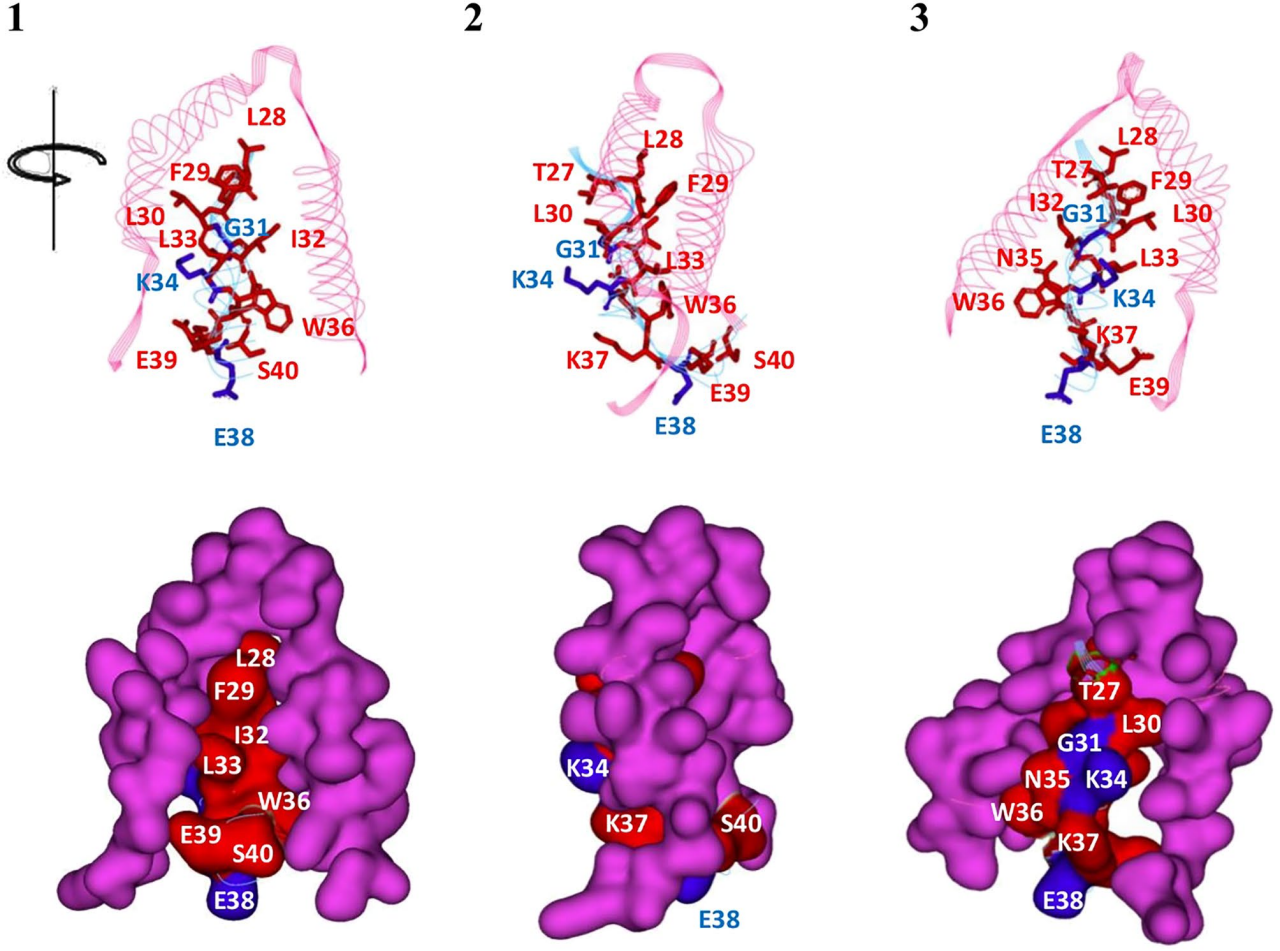
Rotation view of intermolecular neighbors of ^27^TLFLGILKNWKEES^40^ in human IFN-γ. The interactive (red) and non-interactive (blue) residues on IFN-γ chain A (sky) with respect to IFN-γ chain B (pink) were classified by the bond length between two chains being ≤ 5 and 5 Å, respectively.

**Figure 4:**
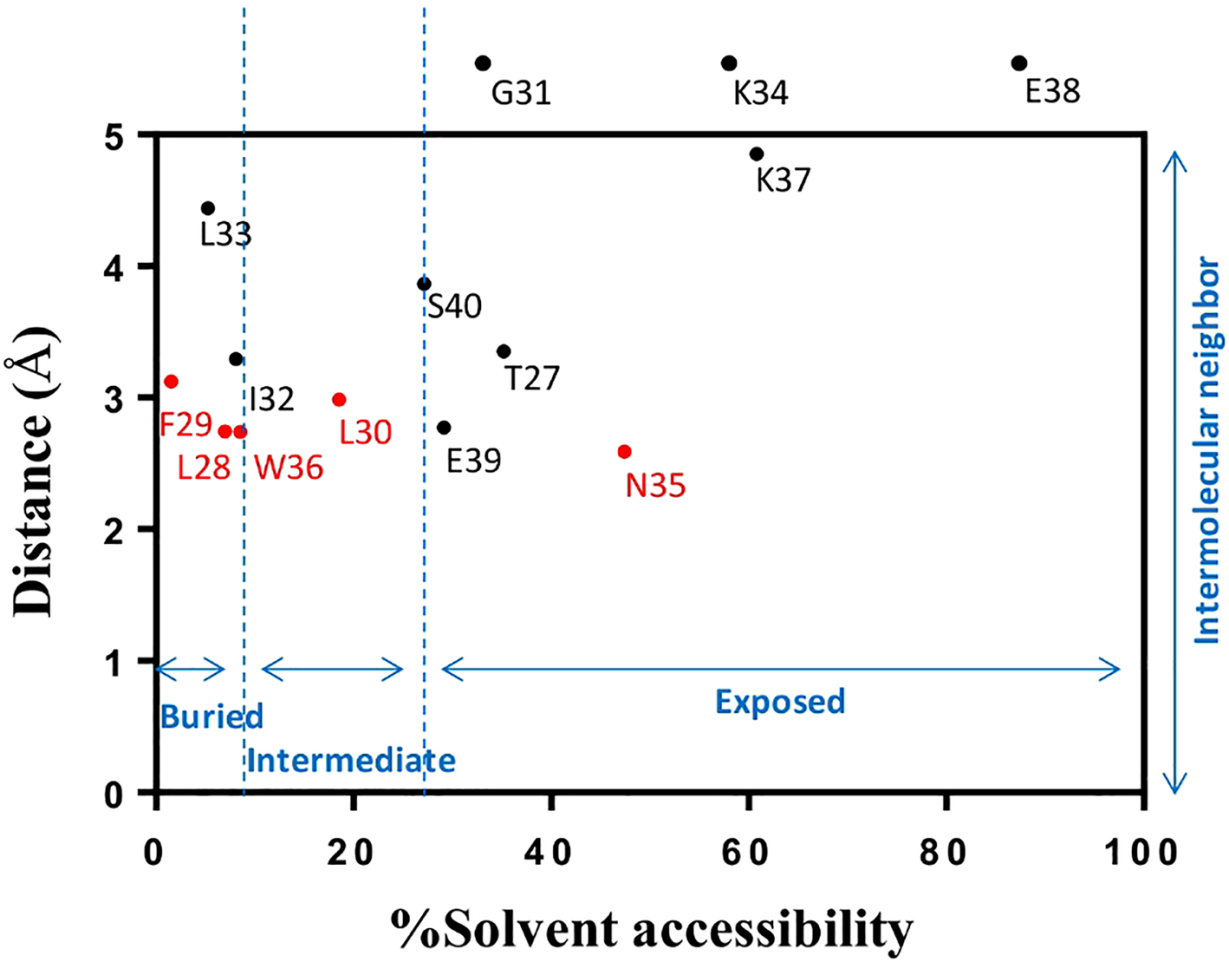
Solvent accessibility, intermolecular neighbors, and hydrogen bond properties of ^27^TLFLGILKNWKEES^40^ in IFN-γ. The red color represents hydrogen bonding.

Based on human IFN-γ homo-dimeric structure (PDB ID: 1FG9), the TDFLRMMLQEER stretch was located in a coil before helix B, in helix B, and the turn between helices B and C (Fig. 1). The gap in the aligned query was at the end of helix B. Focusing on ^27^TLFLGILKNWKEES^40^, the amino acid residues that freely interacted with water and those that interacted with another chain of IFN-γ (referred to as intermolecular residues) were identified based on physicochemical properties, such as solvent accessible surface area (SASA), the binding distance between two IFN-γ chains (at < 5 Å), and intermolecular hydrogen bonding. The SASA calculation revealed that 8, 5, and 1 out of 14 residues were exposed, buried, and intermediate, respectively, as shown in Fig. 2. In terms of interaction with another IFN-γ, 11 residues were considered as intermolecular neighbors (Fig. 3), and five of them were identified as intermolecular hydrogen-bonded neighbors (Fig. 4). As shown in Fig. 4, the combination of these physicochemical properties allowed the classification of residues into three groups, namely (1) the exposed residues interacting with the neighboring IFN-γ molecule (T27, N35, K37, E39, and S40); (2) the exposed residues not interacting with the neighboring IFN-γ molecule (G31, K34, and E38); and (3) the buried residues, including the intermediate ones, interacting with another IFN-γ (L28, F29, L30, I32, L33, and W36).

### Homology modeling of query

**Figure 5:**
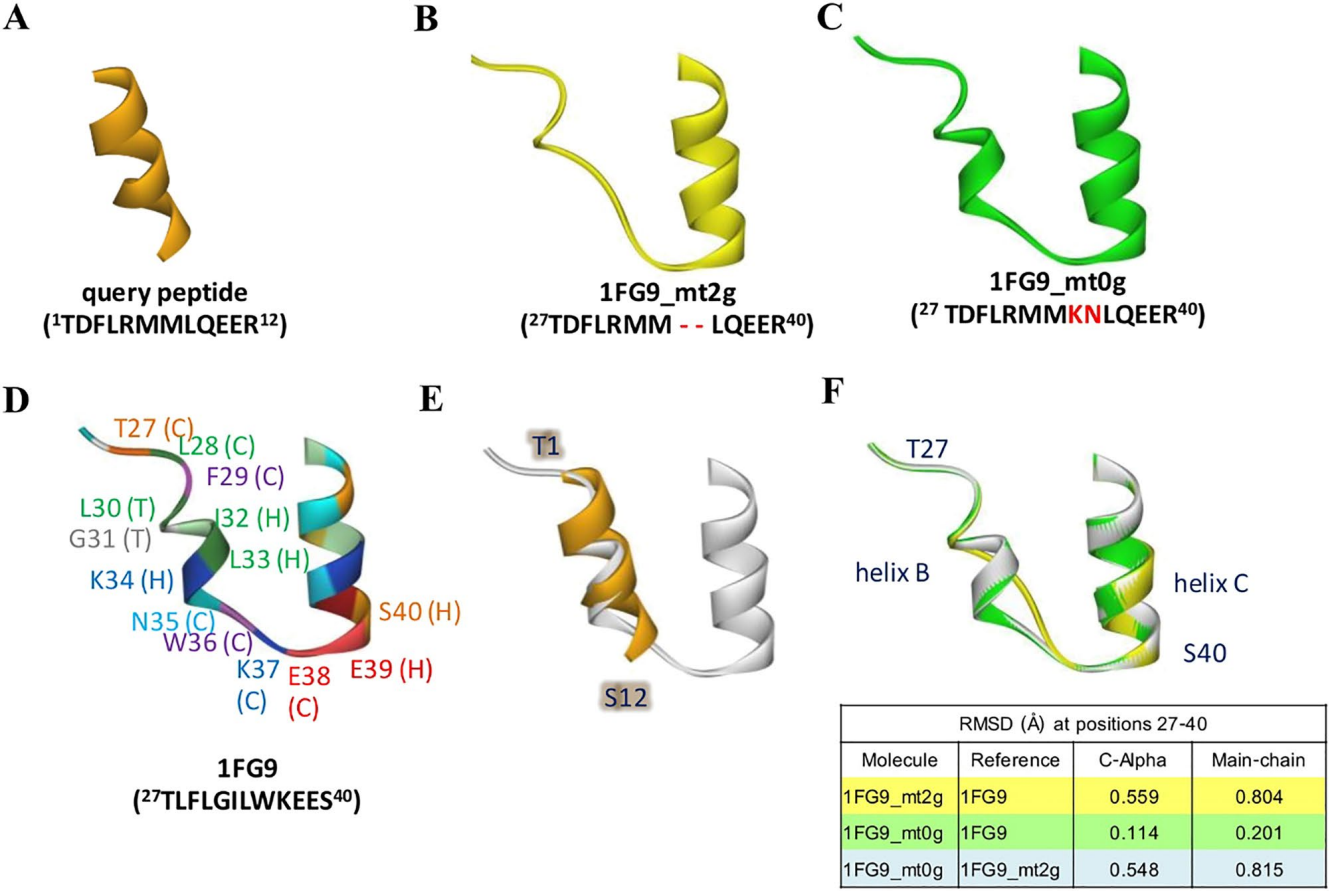
Predictive models of query. (**A**) The query peptide (orange), (**B**) 1FG9_mt2g (yellow), (**C**) 1FG9_mt0g (green), (**D**) 1FG9, (**E**) superimposed structure of the peptide and 1FG9, and (**F**) superimposed structure of 1FG9_mt2g, 1FG9_mt0g, 1FG9, and their RMSD values.

Three different 3D structures of the query were built, using one peptide and two mutated full-length human IFN-γ (PDB ID: 1FG9), as shown in Fig. 5A–C. In Fig. 5E, superimposition of peptide and 1FG9 revealed the 8LQE10 and 11ER12 of the peptide to be helix-forming residues in the same helix, whereas 36WKE38 and 39ES40 of 1FG9 presented coil and helix style, respectively (Fig. 5D). In contrast to the query peptide, the 39ES40 of 1FG9_mt2g and 1FG9_mt0g acted as the initiation of helix C (Fig. 5F), similar to 1FG9. Moreover, at positions 32–33, the 32MM33 of 1FG9_mt2g presented coil style, whereas those of 1FG9_mt0g and 1FG9 were helix type. Considering the query part, the C-alpha RMSD of superimposed structure between 1FG9_mt2g and 1FG9_mt0g was 0.548 Å, which indicated that deletion of 34KN35 affected the secondary structure of helix B.

### Analysis of interaction energies of IFN-γ T27 and A27 with its receptors

According to the physicochemical properties of the B27 mAb-recognized amino acid sequence, threonine at position 27 (T27) was one of the most exposed and an intermolecular residues. From PRODIGY analysis (Supplementary Table 1), at position 27, the critical amino acids of receptor that interact with IFN-γ were found to be TYR49 (Y49), GLY50 (G50), and ASN79 (N79). The energy-minimized structure in the gas phase was used to calculate the interaction energy and investigate the individual residue interaction by comparing the relative values, with lower value indicating a stronger interaction. The binding interface of 5 Å involved the IFN-γ peptide sequence of eight serial amino acid residues, namely ^25^NGTLFLGA^32^/ ^25^NGALFLGA^32^ and the three amino acid residues Y49, G50, and N79 of the receptor. Comparison of the interaction energy of IFN-γ T27 and A27 with its receptor (Table 1) for both individual and interface interactions showed no significant difference, with the value < 1 kcal/mol. Less favorable binding between chain B of IFN-γ and chain D of the receptor was observed (approximately 17 kcal/mol) when threonine at position 27 was changed to alanine. However, the amount of energy had no significant effect on the experimental binding affinity. In agreement with the PRODIGY result, the overall predicted binding affinity (∆G) and dissociation constant (Kd) between wild type and T27A mutant were not different. The predicted ∆G and Kd values from PRODIGY were − 9.5 and − 9.2 kcal/mol and 1.1 and 1.9 × 10^–7^ M at 25.0 °C for wild type (T27) and T27A, respectively.

**Table 1:** Comparison of the interaction energy using AMBER forcefield of IFN-γ T27 and A27 with its receptor. The selected amino acids in 5 Å interface were explored.

IFNG	Receptor	IE (kcal/mol)
**THR27**		
Chain B	Chain D	276.80
ASN25-ALA32	TYR49 GLY50 ASN79	11.74
THR27	TYR49 GLY50 ASN79	4.29
THR27	TYR49	3.02
THR27	GLY50	1.07
THR27	ASN79	0.19
**ALA27**		
Chain B	Chain D	294.09
ASN25-ALA32	TYR49 GLY50 ASN79	11.18
ALA27	TYR49 GLY50 ASN79	3.30
ALA27	TYR49	2.27
ALA27	GLY50	0.96
ALA27	ASN79	0.08

### Binding activity of anti-IFN-γ mAbs to synthetic peptides and their neutralizing activity

**Figure 6:**
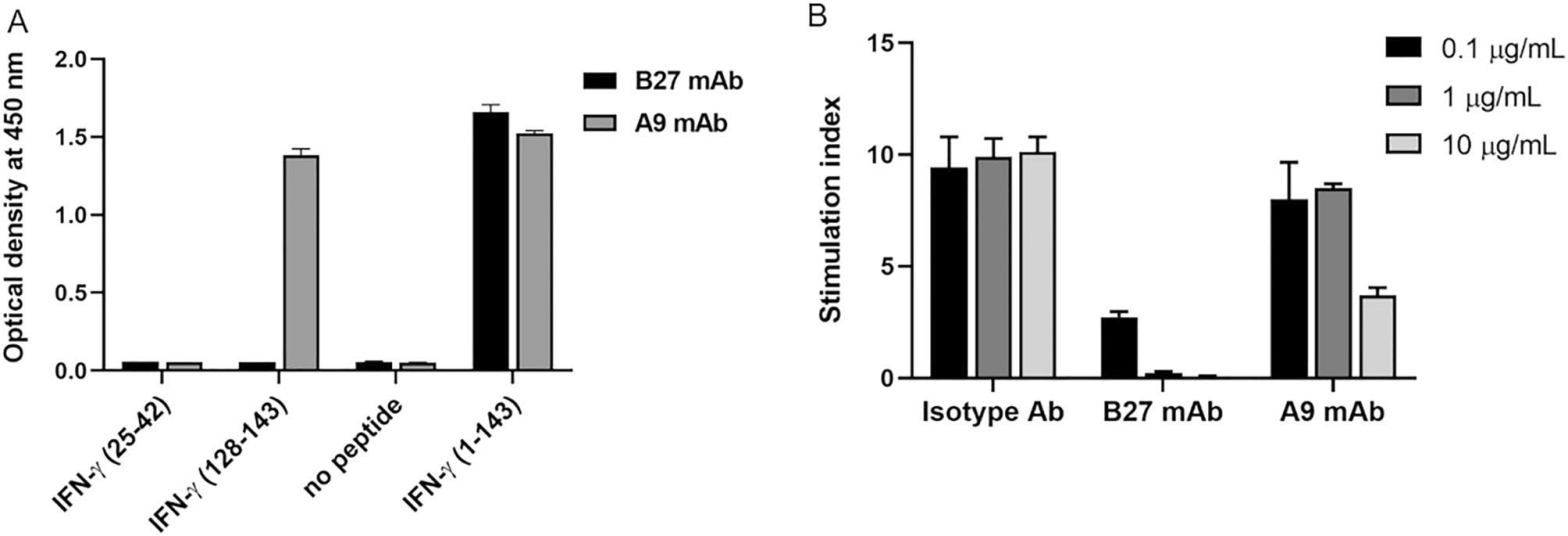
Binding activity of anti-IFN-γ mAbs to the synthetic peptides, and their neutralizing activity. (**A**) Indirect ELISA for the binding of B27 or A9 mAbs to IFN-γ synthetic peptides from residues 25–42 and residues 128–143. (**B**) Neutralizing capacity of B27 and A9 mAbs. The stimulation index was calculated from the expression of MHC class II after IFN-γ stimulation divided by that before stimulation. Three independent experiments were performed, and data are represented as mean ± SD.

Based on sequence alignment, the peptide sequence ^27^TLFLGILKNWKEES^40^ was identified and selected as the target for verifying whether B27 mAb binds to this region. Indirect ELISA was performed, and B27 mAb did not recognize the synthetic peptide target, as shown in Fig. 6A. In addition, the binding activity of A9 mAb against the C-terminal synthetic peptide of IFN-γ was positive. A cell-based functional assay was performed to compare the neutralizing efficiency of mAbs targeting B27 and C-terminal epitopes. Results showed approximately 70% reduction in IFN-γ-mediated MHC class II expression by B27 mAbs at 0.1 µg/mL and almost 100% reduction at 1 µg/mL. However, A9 mAbs demonstrated lesser neutralizing activity with approximately 15% and 60% reduction at 1 and 10 µg/mL concentrations, respectively (Fig. 6B).

### Binding activity of anti-IFN-γ mAbs to IFN-γ wild-type and mutants

**Figure 7:**
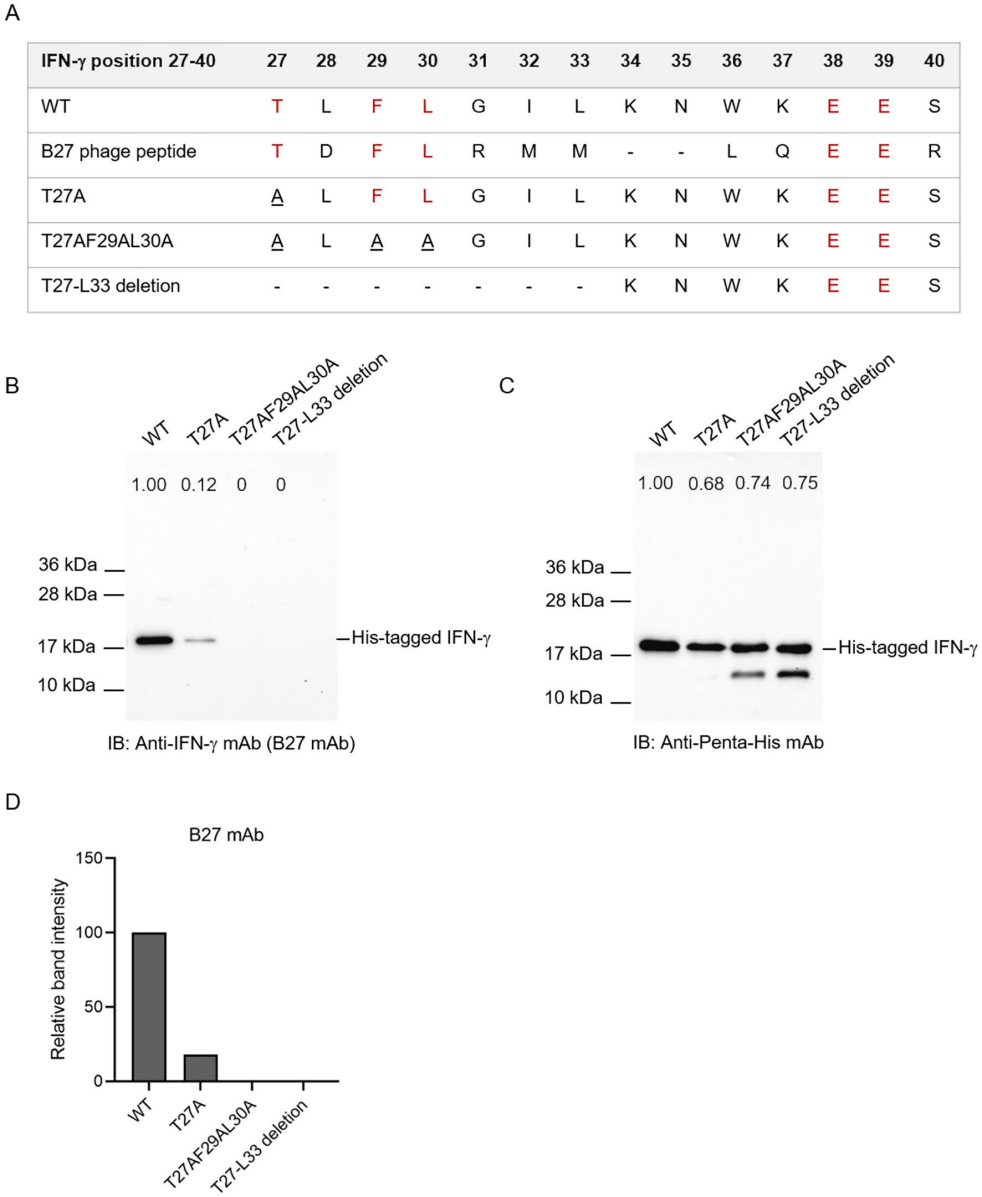
Binding activity of B27 mAb to IFN-γ WT and mutants using western blot analysis. (**A**) The amino acid sequence at positions 27–40 of IFN-γ WT and mutants. (**B**) Western blot analysis of B27 mAb concerning IFN-γ WT, T27A, T27AF29AL30A, and T27-L33 deletion. (**C**) Anti-Penta-His antibody was used to demonstrate the presence of each IFN-γ in bacterial soluble fractions. Band intensity of IFN-γ obtained by B27 mAb and anti-Penta-His antibody was quantified. The number represented the relative level of each IFN-γ mutant to WT. (**D**) Band intensity of IFN-γ detected by B27 mAb was normalized by those achieved by anti-Penta-His antibody. The normalized band intensity of IFN-γ mutants was compared with those in WT. Original blots are presented in Supplementary Figs. 4 and 5.

**Figure 8:**
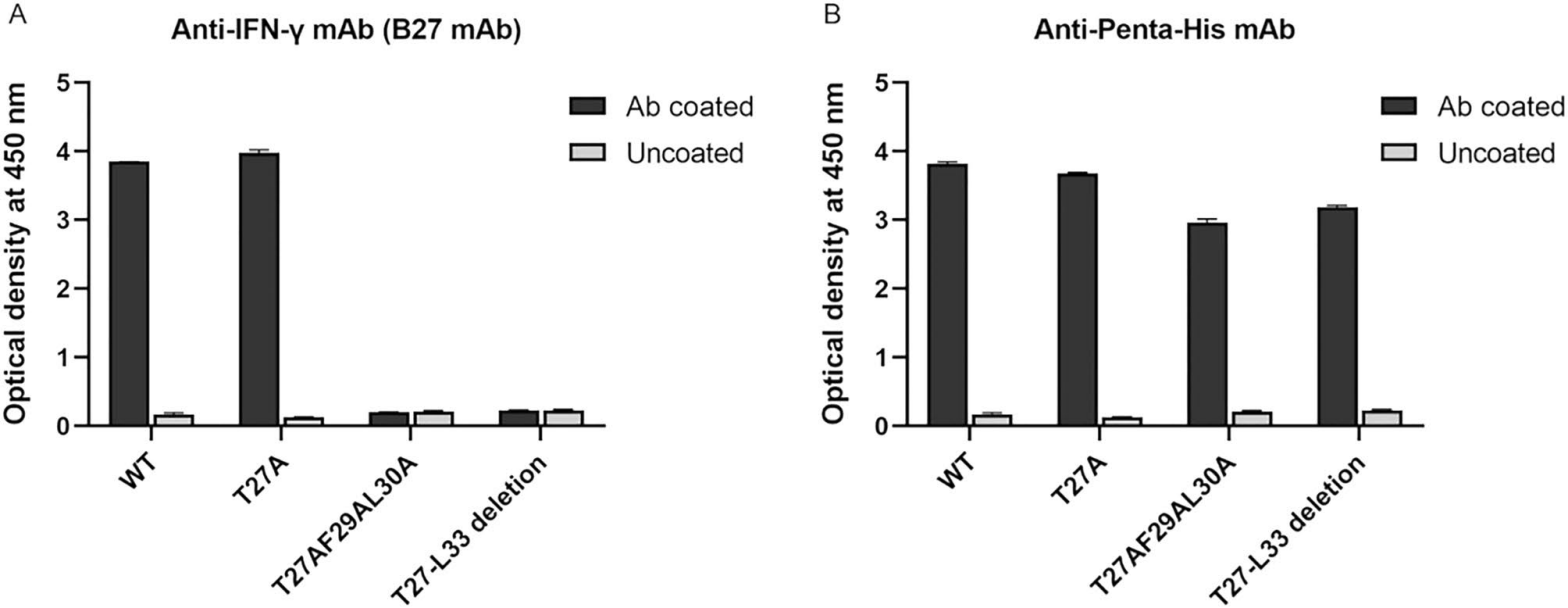
Reactivity of B27 mAb to IFN-γ WT and mutants using sandwich ELISA. (**A**) IFN-γ was captured by B27 mAb and monitored by HRP-conjugated anti-6 × His mAb (**B**) IFN-γ was captured by anti-Penta-His antibody and monitored by HRP-conjugated anti-6 × His mAb. Three technical replicates of all ELISA experiments were performed, and data are shown as mean ± SD.

From the identified amino acid sequence, IFN-γ could be divided into two compartments based on the position of the missing peptide (K34N35); the sequences were TLFLGIL (position 27–33) and WKEES (position 36–40). Since threonine at position 27 was exposed and involved in receptor interaction, TLFLGIL was assumed to participate in B27 mAb binding. To prove this, IFN-γ mutants, including IFN-γ T27A, T27AF29AL30A, and T27–L33 deletion, were generated (Fig. 7A). To determine the binding of B27 mAb to IFN-γ WT and mutants, western blot analysis was performed, and the results demonstrated the binding activity of B27 mAb to IFN-γ T27A to be reduced in contrast to that in wild-type. Simultaneous substitution of T27, F29, and L30 with alanine or deletion of T27–L33 in IFN-γ remarkably deteriorated its antigenicity for B27 mAb (Fig. 7B–D). In addition, the binding of B27 mAb to IFN-γ mutants was also investigated by sandwich ELISA. The results revealed that B27 mAb failed to capture IFN-γ T27AF29AL30A and T27–L33 deletion (Fig. 8A,B).

## Discussion

The presence of anti-IFN-γ autoAbs is tightly associated with severe NTM and intracellular pathogen infections in patients with AOID^6–10^. Disruption of IFN-γ-mediated signaling by neutralizing autoAbs results in immune defects^15^. The IFN-γ-IFN-γR1 interaction is the initial step for IFN-γ-mediated signaling. The regions of IFN-γ involved in receptor binding have been reported to include the loop connecting the A and B helices (residues 18–26), the helix F, and the C-terminal region^3^. Recently, the autoAb against C-terminal epitope was found in patients with mycobacterial infection^11^, which indicated that autoAbs block receptor binding, inhibit IFN-γ signaling, and result in the development of immunodeficiency syndrome. Interestingly, the C-terminal peptide was recognized by autoAbs in only 40% of the 63 AOID cases reported previously^16^. Such findings indicated that either linear or conformational epitope stimulate the production of neutralizing anti-IFN-γ autoAbs. To understand the mechanisms underlying the pathogenesis of AOID better, anti-IFN-γ autoAbs from patients should be characterized. In our previous study, the autoAbs of patients with AOID recognized the same epitope as B27 mAb^12^. Since B27 mAb did not recognize the C-terminal epitope identified by Lin et al.^11^, the epitope for a particular autoAb in patients with AOID was investigated further. A phage display random peptide library was selected to identify the B27 epitope in the present study. Although six phage clones were sequenced, only one peptide “TDFLRMMLQEER” was retrieved, which suggested that B27 mAb has a favorable binding affinity to this particular sequence. The obtained sequence was further analyzed using BLAST and HHblits through the SWISS-MODEL server. The predicted sequence relying on IFN-γ (PDB ID: 1FG9) was ^27^TLFLGILKNWKEES ^40^, located just before helix B, in helix B, and the turn between helices B and C. However, the binding of B27 mAb to this peptide was negative in ELISA, which suggested that the B27 epitope possibly relies on the discontinuous structure of IFN-γ.

As B27 mAb did not bind to the ^27^TLFLGILKNWKEES^40^, T27 mutation (T27A) was introduced and subsequently checked the interaction with B27 mAb using western blot analysis and sandwich ELISA. The results showed that the binding activity of B27 mAb to IFN-γ T27A using western blotting is partially decreased. Whereas the sandwich ELISA demonstrated that the reactivity of B27 mAb against T27A was not significantly changed. It suggested that B27 mAb recognizes the discontinuous structure, which was disrupted in the reducing condition of SDS-PAGE and western blot analysis. Apart from T27, the other residues preserved the structure targeted by the B27 mAb. Since T27 single mutation is not adequate to eradicate this epitope, F29 and L30 are selected for additional modification regarding the predicted bonding interaction between individual amino acids. As shown in Supplementary Figs. 1 and 2, L30 forms bonding with T27, and F29 forms bonding with surrounding amino acids. In contrast, L28 does not interact with other residues among ^27^TLFLGILKNWKEES^40^ (Supplementary Fig. 3). Accordingly, based on the bond interaction network, we hypothesize that T27, F29, and L30 should participate for antigenic determinant. The reactivity of B27 mAb against IFN-γ T27AF29AL30A mutant and T27-L33 deletion was investigated. The reactive band and optical density of these mutants obtained from western blot analysis and sandwich ELISA vanished. It supported that T27, F29, and L30 residues participate in the antigenic determinant recognized by B27 mAb. Regarding the structure analysis of the IFN-γ/receptor complex (PDB ID: 1FG9), this location participates in the interaction. Accordingly, antibodies recognizing this region supposedly neutralize IFN-γ activity.

To further verify the implication of this region in IFN-γ bioactivity, a computer-based analysis was performed. From PRODIGY analysis (Supplementary Table 1), T27 was identified as the critical amino acid interacting with IFN-γR1 at Y49, G50, and N79. The T27 residue was close to the AB connecting loop (position 18–26), which was essential for receptor interaction. The data implied that the autoAb against this region affects the IFN-γ/IFN-γR1 interaction. Regarding the reduction of B27 mAb binding against T27A, the significance of this candidate epitope was confirmed by a null reaction with T27AF29AL30A and T27–L33 deletion. This evidence suggests that these mutations affected the loop structure in IFN-γ. The data implies that B27 mAb interacted favorably with the loop structure. Deletion of T27–L33 was found to affect the interaction energy, although T27AF29AL30A gave results similar to IFN-γ wild-type. Overall, the findings supported the idea that neutralizing antibody specific to the B27 mAb-recognized epitope is crucial for inhibiting cellular signaling of IFN-γ. This evidence supports the hypothesis that the pathology of AOID results from the autoantibody to this epitope.

According to the formerly identified C-terminal epitope^11^, the neutralizing efficiency of A9 mAb, specific to KTGKRKRSQMLFRGRRASQ, and B27 mAb was compared. Results revealed that B27 mAb neutralized IFN-γ activity much more efficiently than A9 mAb at the same concentration. Due to its flexibility, the C-terminal region is not visible in the crystal structure (PDB ID: 1FG9). A previous report had demonstrated that the C-terminal sequence of IFN-γ is a heparin sulfate-binding domain^17^. Heparan binding to IFN-γ resulted in the interference of IFN-γ/IFN-γR1 interaction. Although this region seemed to participate in receptor binding, truncated IFN-γ with deletion of the C-terminal portion did not significantly alter the binding affinity of IFN-γ to IFN-γR1^17^. Consequently, we suggested that A9 mAb interacts with the C-terminal epitope beyond the primary interaction of IFN-γ/IFN-γR1 and partially hinders the occurrence of 2:2:2 IFNγ-IFNγR1-IFNγR2 complex. In contrast, B27 mAb prevents the initial interaction of IFN-γ with IFN-γR1, which is a crucial step for cellular signaling. We hypothesized that the presence of neutralizing antibody against the B27 epitope is involved in the IFN-γ signaling defect. The evidence might mimic the pathogenesis in patients with AOID and hence require further studies.

Apart from autoAbs to IFN-γ, neutralizing Abs are commonly found in patients with anti-cytokine autoAb disease (ACAD)^18^. There are several approaches for identifying the epitopes recognized by anti-cytokine autoAb. For anti-GM-CSF autoAbs in patients with IPAP, the neutralizing epitopes were characterized by the generation of mAbs against GM-CSF from patients. These autoAbs target at least four non-overlapping conformational epitopes on GM-CSF and are dependent on disulfide bond formation^19^. For anti-TNF-α autoAbs in patients with RA, the neutralizing epitopes were investigated using the phage display random peptide library^14^. The evidence suggested that neutralizing autoAbs in ACAD may be generated via different mechanisms and display distinct characteristics. The results revealed that discontinuous epitopes play significant roles in pathogenesis. The phage display technique, in concert with structure-based analysis, presented a promising strategy for the discovery of non-sequential neutralizing epitopes.

Lack of IFN-γ-mediated anti-mycobacterial activity due to anti-IFN-γ autoAbs can cause severe symptoms in patients with AOID. Treatments that restore the IFN-γ functions can be useful to the patients. Replacement of the residues at positions 121–127 (SPAAKTG) of human IFN-γ with the corresponding sequence (LPESSLR) from the mouse has been reported to reduce autoAb binding and increase the bioactivity of IFN-γ in the presence of autoAb^11^. This finding suggested that modification of neutralizing epitopes promotes the escape of IFN-γ from neutralizing autoAbs and enhances IFN-γ-mediated functions. We proposed that the B27 epitope is accessed by a population of neutralizing anti-IFN-γ autoAbs. Therefore, modification of the characterized B27 epitope that hinders autoAb interaction would be valuable as a supplement treatment for patients with AOID who have the autoAbs. Regarding BLAST alignment, the B27 epitope was highly conserved across vertebrates with more than 80% homology. Genetically engineered amino acid variants that retain the immunological activity while reducing immunogenicity will be potential candidates for a therapeutic approach in AOID. Since autoAbs are diverse across individuals, identification of neutralizing epitopes for anti-IFN-γ autoAbs would provide precise diagnosis and treatment. Our study provided data regarding the epitope recognized by B27 mAb relating to autoAbs from patients with AOID. Identifying this novel neutralizing epitope is significant for further deciphering the molecular mechanisms reflecting the AOID pathogenesis.

## Materials and methods

### Epitope mapping by phage display random peptide library

Affinity selection or biopanning process against 12-mer phage display random peptide library (SUT12) was performed, as described previously^20^. Briefly, three rounds of biopanning were performed by reducing the amount of anti-IFN-γ mAbs (clone B27, ImmunoTools, Friesoythe, Germany), from 10, 5, to 2 µg, in each consecutive round of affinity selection. After the first round of biopanning, the eluted phage was amplified overnight. No phage amplification was performed after the second round. Individual phage clones obtained after the third round of biopanning were amplified. Their binding activity against B27 mAb was detected by phage ELISA, as reported previously^21^. To determine the amino acid sequences recognized by B27 mAb in bound phage, phagemids from positive phage clones were prepared. The DNA sequences were determined by automated DNA sequencing services using the -96gII primer (5′-CCC TCA TAG TTA GCG TAA CG-3′). The amino acid sequences were analyzed using SnapGene software.

### Sequence alignment

The query peptide sequence, TDFLRMMLQEER, was aligned with the full-length sequence of human IFN-γ using a pairwise alignment algorithm in BioEdit. Local alignment, known as 'allow end to slide' in BioEdit, with a BLOSUM62 similarity matrix, gap initiation penalty of 8, and gap extension penalty of 2, was used to calculate identity and similarity.

### Structural analysis of human IFN-γ

Specific amino acid residues, related to the query sequence, of the 3D structure of human IFN-γ (PDB ID: 1FG9) were analyzed for their interactive properties, such as water accessibility, intermolecular neighbors, and intermolecular hydrogen bonding. First, the homodimeric structure of IFN-γ was selected to calculate the solvent-accessible surface area (SASA) using an enhanced grid-based numerical algorithm with 240 grid points per atom and a 1.4 Å probe radius. Residues with SASA < 10% were defined as buried residues, whereas those with value beyond the threshold of 25% were called exposed residues. Second, intermolecular neighbors were considered if there were at least two atoms, one belonging to IFN-γ (chain A) and another to IFN-γ (chain B), having a bond distance < 5 Å. Finally, intermolecular bond with distance within 3.5 Å and angles of XDA and DAY within 0 to 180 degrees were used to identify an intermolecular hydrogen bond.

### Homology modeling of query

Comparative and predictive 3D structures of the query were built using three different structures, i.e., one peptide and two mutant IFN-γ molecules. The peptide structure consisted of 12 residues, and its sequence was identical to the query, TDFLRMMLQEER. For mutant forms, the query structures were constructed together with other parts of human IFN-γ. Using the same template structure (PDB ID: 1FG9), the protein sequences were similar to that in the full-length human IFN-γ, except in positions 27–40, which were substituted by ^27^TDFLRMM—LQEER^40^ and ^27^TDFLRMMKNLQEER^40^, referred to as 1FG9_mt2g (127 residues) and 1FG9_0g (129 residues), respectively. K34 and N35 of 1FG9_mt0g were copied from human IFN-γ. To obtain the target-template alignment, different target sequences were employed to search for the template, with BLAST and HHblits using the SWISS-MODEL server. The models were built based on target-1FG9.A alignment using ProMod3.

### Interaction energies of IFN-γ T27 and A27 with its receptors

The 3D structure of human IFN-γ (PDB ID: 1FG9) was downloaded from Protein Data Bank (PDB)^22^. After the removal of water molecules and non-protein molecules, missing atoms were added, and the initial structure was optimized to remove steric clashes using AMBER forcefield in HyperChem 7.5 software package by short minimizations until Root Mean Squared (RMS) gradient tolerance of 0.1000 (kcal/(Å mol)). The amino acids at the position T27 were mutated with UCSF Chimera^23^ and followed by short minizations. The interaction energy (IE) analysis was calculated from IE = E_AB_ − E_A_ − E_B_ where A, B indicated each residue fragment in forming the AB complex. Interaction with the receptor was explored for amino acids in 5 Å vicinity from IFN-γ. In addition, the online prediction tool (PRODIGY, http://milou.science.uu.nl/services/PRODIGY) was used to analyze a contact-based predictor of binding affinity in protein–protein complex ^24^.

### Site-directed mutagenesis

Site-directed mutagenesis was conducted to generate plasmid pET21a IFN-γ T27A using the QuickChange® Lightning Multi Site-Directed Mutagenesis Kit (Stratagene, La Jolla, CA), as per the manufacturer’s instruction. IFN-γ T27A was amplified by PCR using the plasmid pET21a IFN-γ as a template. The primers used were 5′-attcttcaaaatgcctaagaaaagcgctccattatccgctacatctgaatg-3′ and 5′-cattcagatgtagcggataatggagcgcttttcttagcattttgaagaat-3′. The PCR reaction was performed with an initial denaturation step at 95 °C for 2 min, followed by 18 cycles of denaturation at 95 °C for 20 s, annealing at 60 °C for 10 s, and extension at 68 °C for 75 s, and a final extension at 68 °C for 5 min. The PCR product was digested with DpnI at 37 °C for 5 min to eliminate any methylated parental DNA template and transformed into competent *E. coli* strain XL1-Blue. The correct mutant clone was verified by digestion with HindIII and NheI. Colony PCR was subsequently performed with AmpMaster™ Taq Master Mix, GeneAll Biotechnologies, using the primers 5′-gaggaggagaagcttttagtgatggtggtgatggtgaccagaagactgggatgctcttcg-3′ and 5′-gaggaggaggctagcatgcaggacccatatgtaaaagaagcagaaaaccttaagaaa-3′. PCR reaction was conducted with an initial denaturation step at 95 °C for 2 min, followed by 30 cycles of denaturation at 95 °C for 30 s, annealing at 55 °C for 30 s, and extension at 68 °C for 1 min, and a final extension at 68 °C for 5 min. Finally, the plasmid pET21a IFN-γ mutant clone with T27A was extracted from *E. coli* strain XL1-Blue using the QuickGene-Mini80 kit to perform DNA sequencing.

### Cloning of IFN-γ mutants

The cDNA sequence of IFN-γ mutants, including IFN-γ T27AF29AL30A or IFN-γ T27-L33 deletion from pUC57 plasmid, was subcloned into pET21a IFN-γ using NheI and Bsp119I restriction enzymes. After ligation, the pET21a plasmids containing mutated IFN-γ sequence were transformed into competent *E. coli* strain XL1-Blue. Colony PCR was performed using the primers 5′-gaggaggagaagcttttagtgatggtggtgatggtgaccagaagactgggatgctcttcg-3′ and 5′-gaggaggaggctagcatgcaggacccatatgtaaaagaagcagaaaaccttaagaaa-3′. The correct mutant clone was verified by digestion with HindIII and NheI. The plasmid pET21a IFN-γ mutant clones with IFN-γ T27AF29AL30A or IFN-γ T27-L33 deletion were extracted from *E. coli* strain XL1-Blue using the QuickGene-Mini80 kit to perform DNA sequencing.

### Expression of recombinant IFN-γ

The plasmids encoding IFN-γ wild-type (WT) or T27A were transformed into BL21 (DE3) competent cells to produce recombinant IFN-γ protein (rIFN-γ). The cells were grown in 3 mL of super broth (SB) medium at 37 °C overnight and subsequently inoculated in 100 mL of SB medium containing 1% glucose and 100 μg/mL ampicillin at 37 °C. For IFN-γ T27AF29AL30A or T27-L33 deletion, the plasmids were transformed into BL21 (DE3) competent cells harboring chaperone plasmid, pG-KJE8. The cells were cultured in SB medium containing 1% glucose, 100 μg/mL ampicillin, and 20 μg/mL chloramphenicol. Protein expression was induced by adding 1 mM IPTG when the optical density of the culture at 600 nm (OD_600nm_) reached 0.6–0.8, and the culture was continued for 16 h at 30 °C. The induced cells expressing rIFN-γ were washed with phosphate-buffered saline (PBS), lysed by freeze–thaw with 5-min sonication thrice, followed by centrifugation at 15,000× *g*, 4 °C for 30 min. The soluble fractions were collected for western blotting and sandwich ELISA.

### Indirect ELISA

Indirect ELISA was performed to verify whether anti-IFN-γ mAb (clone B27) binds to IFN-γ position 27–40. Microtiter plates were coated with 50 μL of streptavidin (2 μg/mL) in bicarbonate buffer (pH 9.6) per well and incubated overnight at 4 °C in a moist chamber. The other steps were performed at 37 °C in a humidified chamber. The coated wells were washed four times with 0.05% Tween 20 in PBS. Peptide ^25^NGTLFLGILKNWKEESDR^42^ labeled with biotin (2 μg/mL) was added and incubated for 1 h. After washing, non-specific binding was blocked with a blocking solution (2% skimmed milk in PBS) for 1 h, and 50 μL of B27 mAb (0.5 μg/mL) was added and incubated for 1 h. After washing four times, 50 μL of HRP-conjugated goat anti-mouse immunoglobulin (dilution 1:3,000) was added and incubated for 1 h. The reactions were developed with TMB substrate and stopped with 1 N HCl. Absorbance was measured at 450 nm with an ELISA reader. In this experiment, anti-IFN-γ mAb (clone A9, Santa Cruz Biotechnology, CA, USA), which recognizes IFN-γ at positions 125–143, was used as the positive control of the detection system.

### Sandwich ELISA

To test the binding activity of B27 mAb to IFN-γ, the plates were coated with B27 mAb (5 μg/mL, 50 μL per well) in bicarbonate buffer overnight at 4 °C. Mouse anti-Penta-His antibody (Qiagen, Germany) was coated into separating wells for indicating the presence of IFN-γ in the soluble fractions. After washing, non-specific protein binding was blocked with 2% skimmed milk in PBS. Bacterial soluble fractions containing IFN-γ WT, T27A, T27AF29AL30A, or T27-L33 deletion (dilution 1:10, 50 μL per well) were added to wells and incubated for 1 h at room temperature. The wells were washed four times with 0.05% Tween 20 in PBS before the addition of horseradish peroxidase (HRP)-conjugated anti-6 × His mAb (BioLegend, San Diego, CA). After 1 h incubation, plates were washed four times, and TMB substrate was subsequently added. The reaction was stopped by adding 1 N HCl, and absorbance at 450 nm was determined using an ELISA microplate reader.

### Western blot analysis

The bacterial soluble fractions containing IFN-γ WT or mutants were subjected to SDS-PAGE under reducing conditions and then transferred to nitrocellulose membrane. The membrane was blocked with 5% skimmed milk in PBS for 1 h at room temperature. Mouse anti-IFN-γ mAb, clone B27 (1 µg/mL), or mouse anti-Penta-His antibody (1 µg/mL) were separately added and incubated with the membrane for 1 h at room temperature with shaking. After washing, the membranes were incubated with an HRP-conjugated goat anti-mouse immunoglobulin antibody (dilution 1:3,000 in 2% skimmed milk in PBS) for 1 h. The membranes were washed, and bands were enhanced using Supersignal™ West Pico Chemiluminescence Substrate (Thermo Fisher Scientific, Waltham, MA, USA); the protein bands were visualized under a ChemiDoc™ MP imaging system (Bio-Rad, France).

### Assessment of the neutralizing activity of anti-IFN-γ mAbs

A cell-based assay was performed to determine the neutralizing activity of B27 and A9 mAbs. Briefly, 10 ng/mL of rIFN-γ WT was incubated with mAb at 0.1, 1, or 10 µg/mL for 1 h. The mixture was subsequently incubated with THP-1 cells (4 × 10^5^ cells) at 37 °C in 5% CO_2_ incubator. After 24 h, cells were harvested to detect MHC class II surface expression by flow cytometry. Cells were washed thrice with PBS and blocked with 50 µL of 10% AB serum in PBS for 30 min. For MHC class II staining, 2.5 µL of FITC-conjugated anti-human HLA-DR and -DP (clone HL-38) or isotype-matched control (FITC-conjugated mouse IgG2a) (ImmunoTools, Friesoythe, Germany) was added and incubated for 30 min on ice. After washing, cells were resuspended in 1% paraformaldehyde-PBS. Data were collected with BD Accuri™ C6 Plus Flow Cytometer (BD Bioscience).

## Supplementary Information

Supplementary Figures.

Supplementary Tables.

## Abbreviations

Anti-IFN-γ autoAbs: Anti-interferon gamma autoantibodies
B27 mAb: Monoclonal antibody clone B27
MHC: Major histocompatibility complex
AOID: Adult-onset immunodeficiency
NTM: Nontuberculous mycobacteria
RA: Rheumatoid arthritis
IFN-γR1: Interferon gamma receptor 1
IFN-γR2: Interferon gamma receptor 2
