# Supplementary material for: Determination of a distinguished interferon gamma epitope recognized by monoclonal antibody relating to autoantibody associated immunodeficiency
Source: Sci Rep. 2022 May 9;12:7608. doi: 10.1038/s41598-022-11774-9 (PMC9085737; doi:10.1038/s41598-022-11774-9)
Supplement: Supplementary file 1 — Supplementary Figures. [file 41598_2022_11774_MOESM1_ESM.pdf]

Supplementary Figures

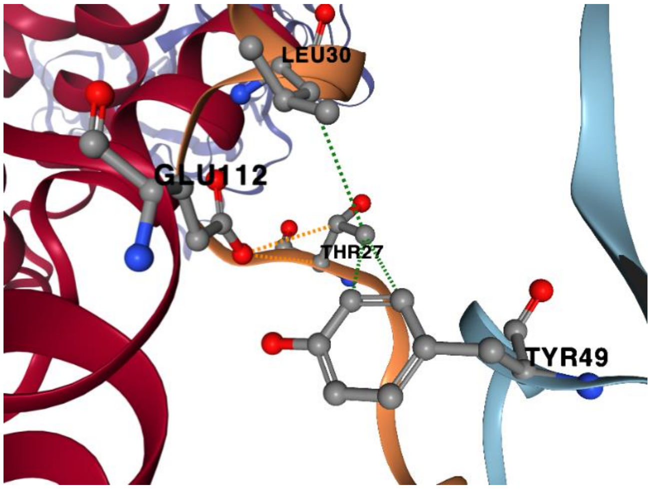

Supplementary Figure 1 Molecular bonding of IFN-γ THR27 (T27) and LEU30 (L30) of 1FG9 PDB complex. Chain A and B of IFN-γ are shown in orange and red, respectively. Blue designated chain D of IFN-γ receptor.

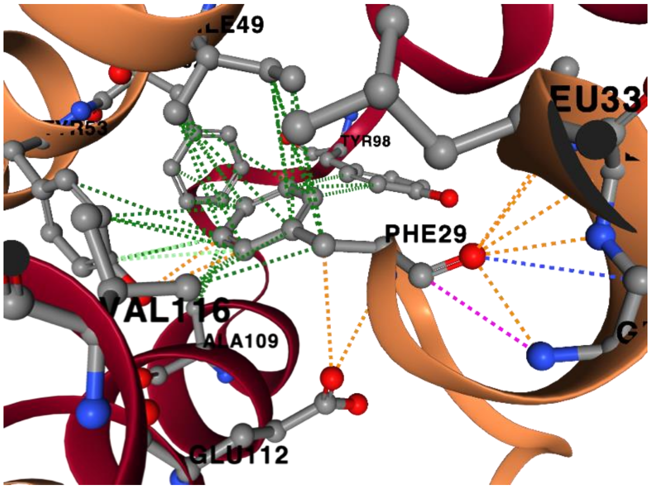

Supplementary Figure 2 Molecular bonding of IFN-γ PHE29 (F29) of 1FG9 PDB complex. Chain A and B of IFN-γ are shown in orange and red, respectively.

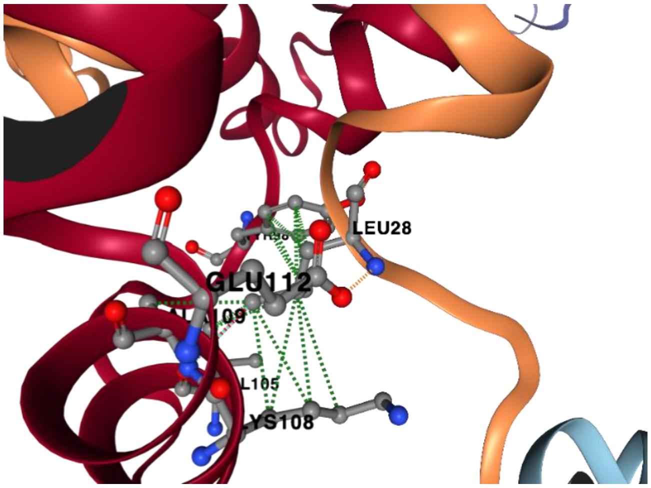

Supplementary Figure 3 Molecular bonding of IFN-γ LEU28 (L28) of 1FG9 PDB complex. Chain A and B of IFN-γ are shown in orange and red, respectively. Blue designated chain D of IFN-γ receptor.

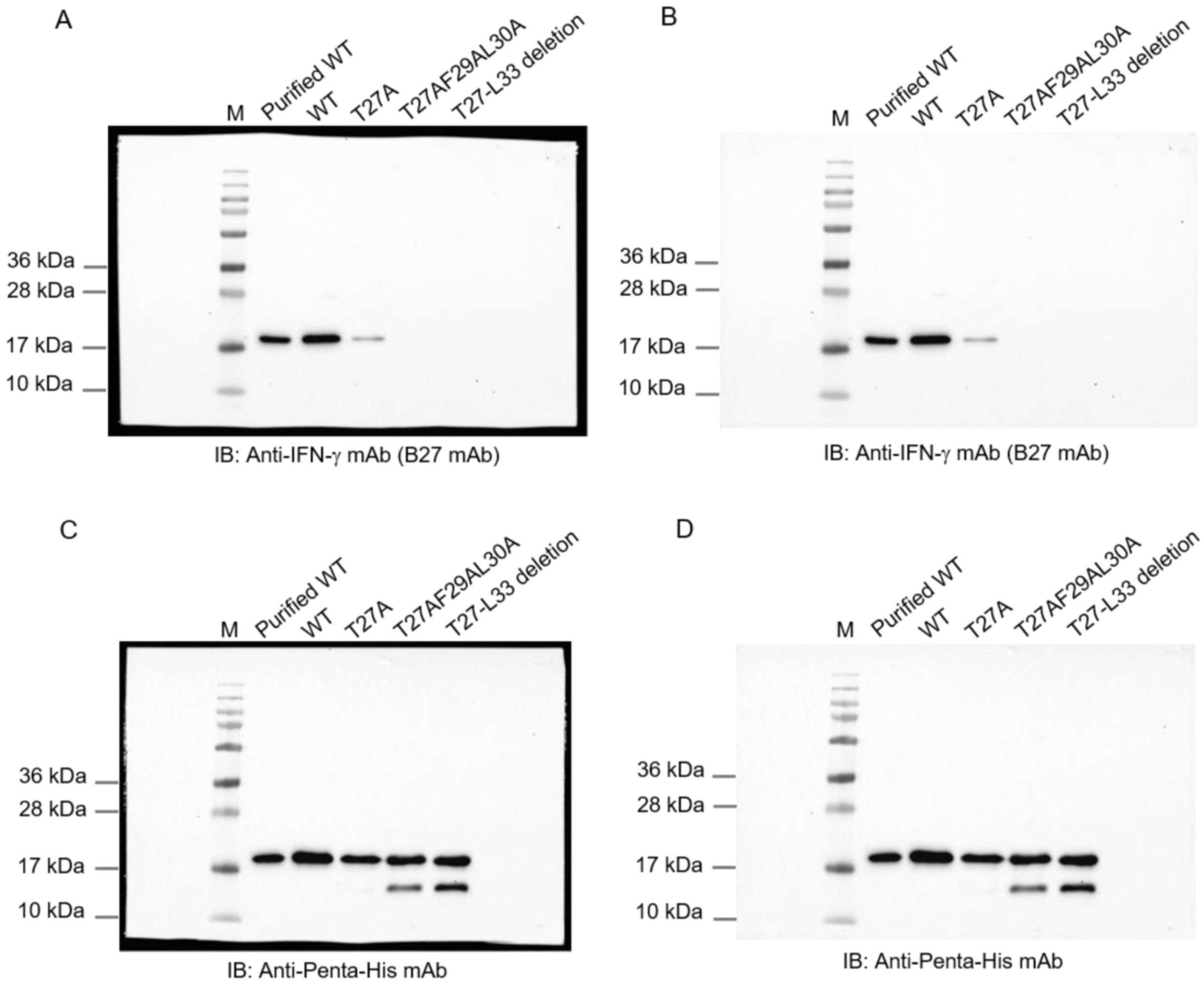

Supplementary Figure 4 Binding activity of B27 mAb to IFN-γ WT and mutants. **(A, B)** Western blotting of B27 mAb with respect to purified IFN-γ WT, the soluble fractions containing IFN-γ WT, T27A, T27AF29AL30A, and T27-L33 deletion, respectively. **(C, D)** Anti-His tag antibody was used for indicating the IFN-γ in each soluble fraction.

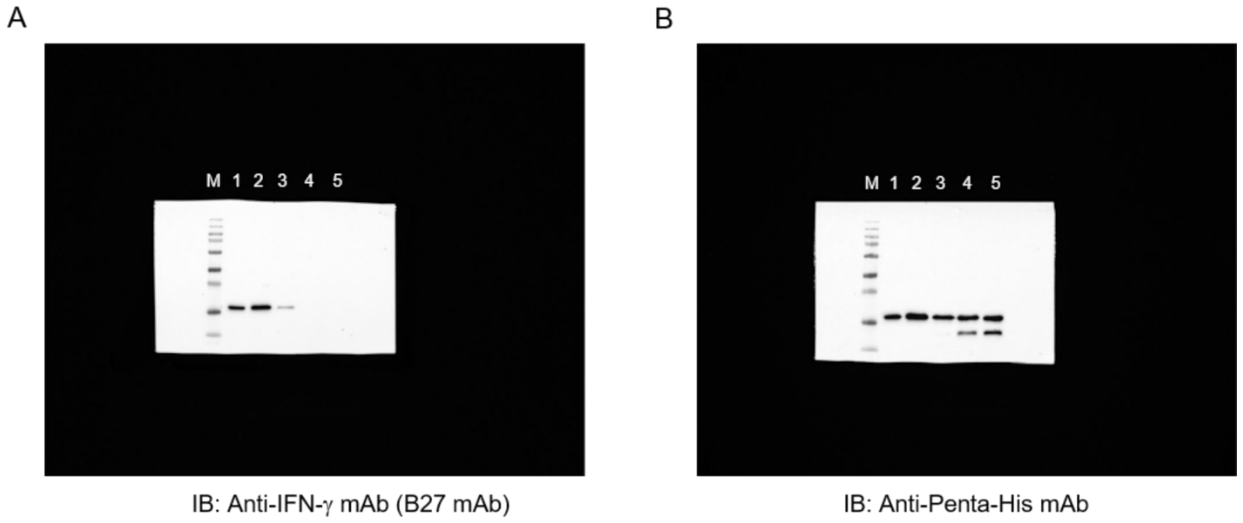

Supplementary Figure 5 Raw image of western blotting results from the ChemiDoc^{TM} MP imaging system. **(A)** Protein bands obtained by B27 mAb reaction **(B)** Protein bands obtained by staining with anti-Penta-His antibody. M, protein molecular weight marker; lane 1, purified IFN-γ WT; lane 2-5, IFN-γ from bacterial soluble fractions including WT, T27A, T27AF29AL30A, and T27-L33 deletion, respectively.
