# Supplementary material for: Determination of a distinguished interferon gamma epitope recognized by monoclonal antibody relating to autoantibody associated immunodeficiency
Source: Sci Rep. 2022 May 9;12:7608. doi: 10.1038/s41598-022-11774-9 (PMC9085737; doi:10.1038/s41598-022-11774-9)
Supplement: Supplementary file 2 — Supplementary Tables. [file 41598_2022_11774_MOESM2_ESM.pdf]

Supplementary Table

Table S1 List of residue-residue pairs at the interface

**WT**	**T27A**
ALA 17 B GLU 101 D	ALA 17 B GLU 101 D
ALA 17 B TRP 82 D	ALA 17 B TRP 82 D
ALA 23 B ASN 48 D	ALA 23 B ASN 48 D
ALA 23 B ASN 53 D	ALA 23 B ASN 53 D
ALA 23 B GLY 50 D	ALA 23 B GLY 50 D
ALA 23 B LYS 47 D	ALA 23 B LYS 47 D
ALA 23 B LYS 52 D	ALA 23 B LYS 52 D
ALA 23 B TRP 82 D	ALA 23 B TRP 82 D
ALA 23 B TYR 49 D	ALA 23 B TYR 49 D
ALA 23 B VAL 51 D	ALA 23 B VAL 51 D
ASN 25 B ASN 53 D	ASN 25 B ASN 53 D
ASN 25 B GLY 50 D	ASN 25 B GLY 50 D
ASN 25 B LYS 52 D	ASN 25 B LYS 52 D
ASN 25 B SER 54 D	ASN 25 B SER 54 D
ASN 25 B VAL 51 D	ASN 25 B VAL 51 D

ASP	2	B	VAL	206	D	ASP	2	B	VAL	206	D
ASP	21	B	LYS	47	D	ASP	21	B	LYS	47	D
ASP	21	B	SER	54	D	ASP	21	B	SER	54	D
ASP	24	B	ASN	53	D	ASP	24	B	ASN	53	D
ASP	24	B	GLU	55	D	ASP	24	B	GLU	55	D
ASP	24	B	GLY	50	D	ASP	24	B	GLY	50	D
ASP	24	B	LYS	47	D	ASP	24	B	LYS	47	D
ASP	24	B	LYS	52	D	ASP	24	B	LYS	52	D
ASP	24	B	SER	54	D	ASP	24	B	SER	54	D
ASP	24	B	VAL	51	D	ASP	24	B	VAL	51	D
GLN	1	B	ARG	106	D	GLN	1	B	ARG	106	D
GLN	1	B	GLY	208	D	GLN	1	B	GLY	208	D
GLN	1	B	TRP	207	D	GLN	1	B	TRP	207	D
GLN	1	B	VAL	206	D	GLN	1	B	VAL	206	D
GLU	9	B	ARG	106	D	GLU	9	B	ARG	106	D
GLU	9	B	TRP	207	D	GLU	9	B	TRP	207	D
GLY	18	B	GLU	101	D	GLY	18	B	GLU	101	D
GLY	18	B	LYS	98	D	GLY	18	B	LYS	98	D
GLY	18	B	TRP	82	D	GLY	18	B	TRP	82	D

GLY	26	B	ASN	53	D	GLY	26	B	ASN	53	D
GLY	26	B	GLY	50	D	GLY	26	B	GLY	50	D
GLY	26	B	LYS	52	D	GLY	26	B	LYS	52	D
GLY	26	B	TYR	49	D	GLY	26	B	TYR	49	D
GLY	26	B	VAL	51	D	GLY	26	B	ASN	53	D
HIS	19	B	LYS	98	D	HIS	19	B	LYS	98	D
HIS	19	B	TRP	82	D	HIS	19	B	TRP	82	D
LEU	28	B	TYR	49	D	LEU	28	B	TYR	49	D
LEU	30	B	ASN	79	D	LEU	30	B	ASN	79	D
LEU	30	B	TYR	49	D	LEU	30	B	TYR	49	D
LYS	12	B	GLU	101	D	LYS	12	B	GLU	101	D
LYS	34	B	ASP	76	D	LYS	34	B	ASP	76	D
LYS	34	B	THR	148	D	LYS	34	B	THR	148	D
MET	0	B	GLY	208	D	MET	0	B	GLY	208	D
MET	0	B	TRP	207	D	MET	0	B	TRP	207	D
SER	20	B	LYS	47	D	SER	20	B	LYS	47	D
SER	20	B	LYS	98	D	SER	20	B	LYS	98	D
SER	20	B	TRP	56	D	SER	20	B	TRP	56	D
SER	20	B	TRP	82	D	SER	20	B	TRP	82	D

THR 27 B ASN 79 D	ALA 27 B GLY 50 D
THR 27 B GLY 50 D	ALA 27 B TYR 49 D
THR 27 B TYR 49 D	
TYR 4 B VAL 206 D	TYR 4 B VAL 206 D
VAL 5 B TRP 207 D	VAL 5 B TRP 207 D
VAL 5 B VAL 206 D	VAL 5 B VAL 206 D
VAL 22 B GLY 50 D	VAL 22 B GLY 50 D
VAL 22 B TRP 82 D	VAL 22 B TRP 82 D
VAL 22 B TYR 49 D	VAL 22 B TYR 49 D
